# Basics of hip arthroscopy: Step‐by‐step technique

**DOI:** 10.1002/jeo2.12021

**Published:** 2024-04-12

**Authors:** Safa Gursoy, Yigit Umur Cirdi, Muge Kirac, Jorge Chahla

**Affiliations:** ^1^ Department of Orthopaedics and Traumatology, Faculty of Medicine Acibadem Mehmet Ali Aydinlar University Istanbul Turkey; ^2^ Department of Orthopaedics and Traumatology Acibadem Atasehir Hospital Istanbul Turkey; ^3^ Department of Orthopaedic Surgery Rush University Chicago Illinois USA

**Keywords:** capsular management, femoroacetabular impingement, hip arthroscopy, osteochondroplasty

## Abstract

Hip arthroscopy is a surgical procedure that has a technically challenging nature, requiring advanced spatial skills and specialised instrumentation. The most common indication for hip arthroscopy is femoroacetabular impingement, which is increasing due to improved awareness and knowledge of the condition among healthcare professionals. Hip arthroscopy requires many different checkpoints from patient positioning to capsule closure to be successfully completed. Patient positioning is one of the keystones of hip arthroscopy and the probability of a surgeon achieving successful outcomes is significantly influenced by the establishment of optimal access points. The importance of the acetabular labrum and capsule has been better understood in recent years. There has been a noticeable preference towards prioritising acetabular labral repair over debridement or excision. Similarly, consistent with the literature, capsule closure restores naive hip biomechanics more successfully and improves functional outcomes following hip arthroscopy. Osteochondroplasty is a frequently employed therapeutic intervention; yet, attaining optimal osteochondroplasty outcomes might present challenges. The aim is, to restore the full perfect sphericity of the femoral head without attenuation of the head. The aim of this article is to highlight the knowledge accumulated from experiences based on previous hip arthroscopy surgeries as a solution for future troubleshooting steps.

**Level of Evidence**: Level V.

**Abbreviations**
AL: anterolateral
ASIS: anterior superior iliac spine
CLJ: chondrolabral Junction
CT: computed tomography
DALA: distal anterolateral accessory
FADIR: flexion, adduction and internal rotation
FAI: femoroacetabular impingement
GT: greater trochanter
HHS: Harris Hip Score
HOS‐ADL: Hip Outcome Score—Activity of Daily Living
LCEA: lateral centre edge angle
mHHS: modified Harris Hip Score
mMA: modified mid‐anterior
NAHS: nonarthritic Hip Score
SF‐12: Short Form ‐12
VAS: Visual Analogue Scale

## INTRODUCTION

The first article on hip arthroscopy was published by Burman in 1931 [7]. He applied experimental arthroscopy techniques on cadaver hips. The era of modern hip arthroscopy procedures started in the late 1980s [8]. In the late 1970s, the first modern hip arthroscopy procedures were performed. The concept of femoroacetabular impingement (FAI) was initially introduced by Ganz et al. [32]. Modern hip arthroscopy has the capability to address both the hip joint and extra‐articular region. However, by far the most common indication for hip arthroscopy is FAI. The prevalence of FAI is increasing due to improved awareness and knowledge of the condition among healthcare professionals [18]. Additionally, comparing conservative treatment and hip arthroscopy for patients with FAI, it was shown that arthroscopy resulted in superior patient‐assessed functional outcomes [14, 30].

Despite the advancements in equipment and traction techniques during the 1990s, hip arthroscopy has not gained the same level of popularity as knee, shoulder, elbow and ankle arthroscopy due to its technically challenging surgical procedures. First of all, the amount of time that will be used for the process is constrained because the traction is applied to the joint and the surgeon must possess advanced three‐dimensional spatial skills to successfully perform the surgery. Also, specialised instrumentation is necessary for the procedure, which adds to its complexity. Lastly, it is important to note that hip arthroscopy is a relatively new surgical technique, which contributes to its demanding nature [5, 13, 22, 31, 34]. Studies have indicated that surgical cases conducted by surgeons who handle a substantial number of cases are associated with a notably reduced likelihood of requiring revision hip surgery, in comparison to cases performed by physicians with lower case numbers [29, 35]. Hip arthroscopy requires many different checkpoints to be successfully completed starting with the positioning of the patient followed by opening accurate portals for optimal access points to the hip joint throughout the surgery. For these reasons, hip arthroscopy is thought to have a steep learning curve.

The awareness of the acetabular labrum has significantly increased in recent years due to progress in arthroscopic hip surgery. There has been a noticeable preference towards prioritising acetabular labral repair over debridement or excision, wherever feasible, because of its significant contribution to maintaining the integrity of the hip joint. Indeed, in instances where labrum repair is not achievable, reconstruction procedures are performed to restore the structural integrity of the labrum [40]. A study conducted by Larson et al. [27] demonstrated that the process of labral refixation yielded improved outcomes in terms of the Harris Hip Score (HHS), Short Form‐12 (SF‐12) and Visual Analogue Scale (VAS) scores at an average follow‐up period of 3.5 years. The findings of a study published by Domb et al. [9] revealed significant improvements in patients’ ratings on the modified Harris Hip Score (mHHS), nonarthritic Hip Score (NAHS), Hip Outcome Score—Activity of Daily Living (HOS‐ADL), VAS and patient satisfaction when compared with their preoperative scores at a minimum 5‐year follow‐up period. Another study conducted by Larson et al. [26] with a follow‐up period of more than 7 years indicated that the cohort who had labral repair/refixation had superior patient‐reported outcomes and reduced rates of failure. The excision/debridement group had notably higher failure rates as time progressed, while the repair/refixation group demonstrated more consistent preservation of good to outstanding outcomes. In contrast to the knee and shoulder, capsulotomy is one of the essential parts of hip arthroscopy for a broader surgical field of view.

The aim of this review is to highlight the knowledge accumulated from experiences based on previous hip arthroscopy surgeries as a solution for future troubleshooting steps.

## STEP‐BY‐STEP TECHNIQUE

### Patient setup

Following the implementation of appropriate protocols for patient identification and safety, the patient is transferred to the designated operating room and positioned on the operating table. General anaesthesia with muscle paralysis is administered. In addition, adjuvant postoperative pain management can be facilitated through the application of regional anaesthetic procedures, including spinal, epidural or lumbar plexus blocks. The hip arthroscopy procedure has been described using both the lateral and supine positions. In our institution, we prefer to operate in the supine position. The patient is placed in a supine posture on an operating table and a lateralized perineal post is fitted under general anaesthesia. The arm on the ipsilateral to operative extremity is appropriately cushioned and positioned across the patient's chest. To minimise the risk of pudendal nerve compression, it is important to ensure that the padding of the perineal post is well cushioned. Applying continuous traction during the hip arthroscopy carries potential risks. It has been shown that traction‐related complications following hip arthroscopy may reach up to 74% of the cases [11]. The majority of the cases are neuropraxia and resolve spontaneously [3].

Postless systems are designed to eliminate traction and pressure‐related complications. Commercially available pads are an alternative to eliminate the risk of iatrogenic injuries in the groin and perineal regions. The use of the pad positioning device can effectively facilitate patient distraction and maintain proper patient placement during the entire surgery. It prevents patient movement by generating friction between the pad and the patient's skin, as well as between the pad and a conventional hip arthroscopy table [36].

**Figure 1:**
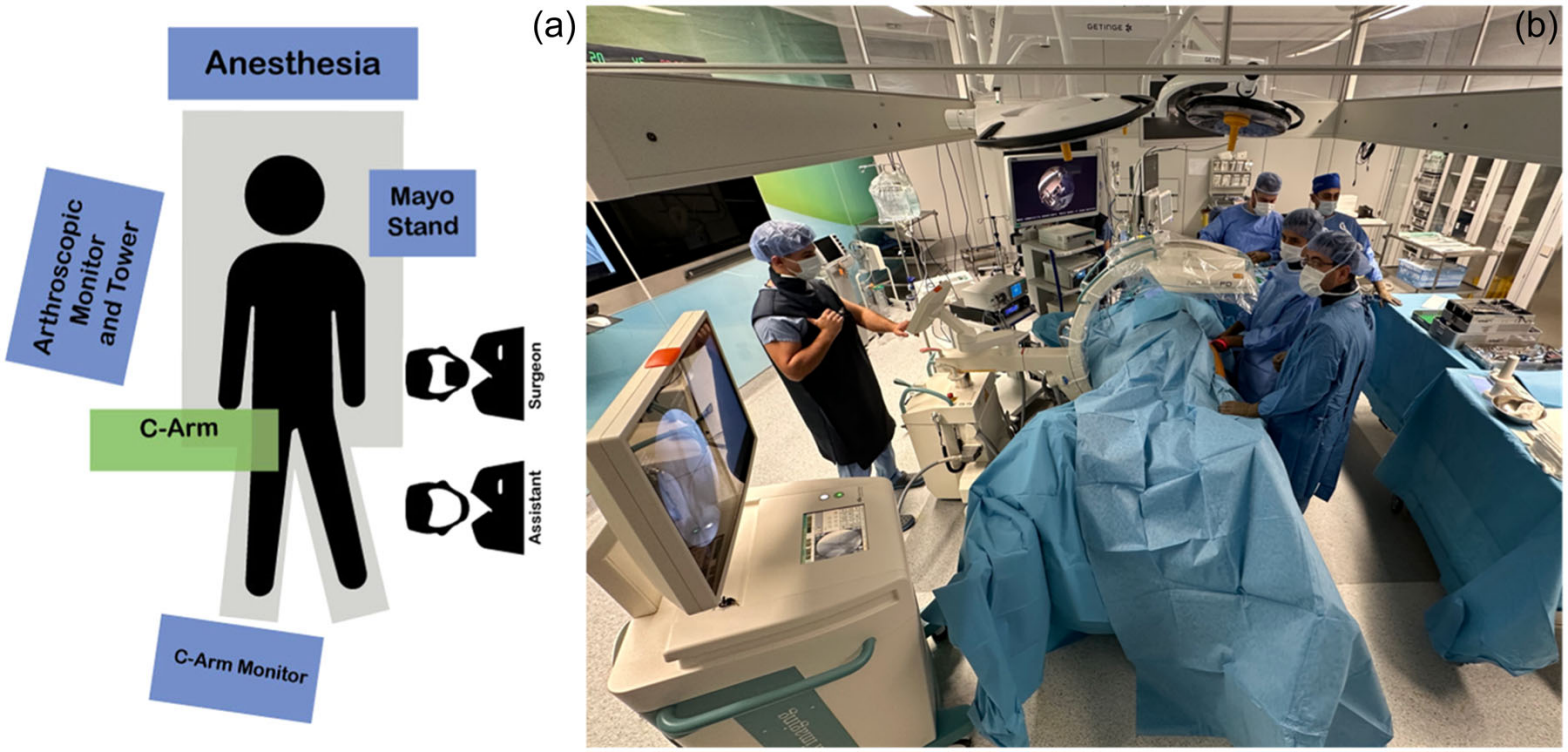
(a) Illustration of an operating room setup for a left‐sided patient. (b) Optimal positioning inside the operating room for a left‐sided patient. The chief surgeon must have an unobstructed vision to both the arthroscopic monitor and C‐arm visualizer at the same time.

Subsequently, the patient's ankles and feet on both sides are inserted into foam‐padded boots. Sufficient space should be provided so the circulating nurse can approach the foot. To gain access to the different areas of the intra‐articular and extra‐articular hip joints, it is required to carry out intraoperative modifications, which involve manipulating the operative extremities. These manipulations may involve the application or removal of traction, as well as the abduction and adduction of the limb, and the flexion and extension of the hip (Figure 1).

#### Protips

- Patient positioning is the keystone. Premature release of the traction may cause catastrophic consequences, such as instrument breakage and chondral damage. Removal of those broken fragments requires excessive effort.
- Multilayered and slightly over‐tight coban wrapping provides adequate hold during the traction time.
- Before proceeding with the CAM resection, partial release of the coban wrap prevents the neurologic complications associated with over‐squeezing over the feet.
- To prevent any nerve injury, keep traction time below 2 h [3].

### Portals and access

Traction manoeuvres require the utmost attention to perform. First, traction is applied while the lower extremity is abducted to provide axial traction. Then, adduction is applied to provide lateral distraction of the hip joint. An anteroposterior view of the hip joint is obtained. Usually, 8–10 mm distraction is adequate. Lastly, slight internal rotation is applied to eliminate anteversion of the proximal femur for a horizontal workspace. Traction force is expected to be higher in male patients, particularly in muscular men and in patients with acetabular over‐coverage. Air venting provides a practical solution to overcome the tremendous amount of force required to provide distraction. A spinal needle is inserted through the capsule and the bouncing sensation of the capsule is obtained. Then, the capsule is punctured and inflated (60cc with an 18 gauge needle). When the positive pressure air applied inside to capsule, negative pressure is eliminated and desired distraction is achieved [36].

**Figure 2:**
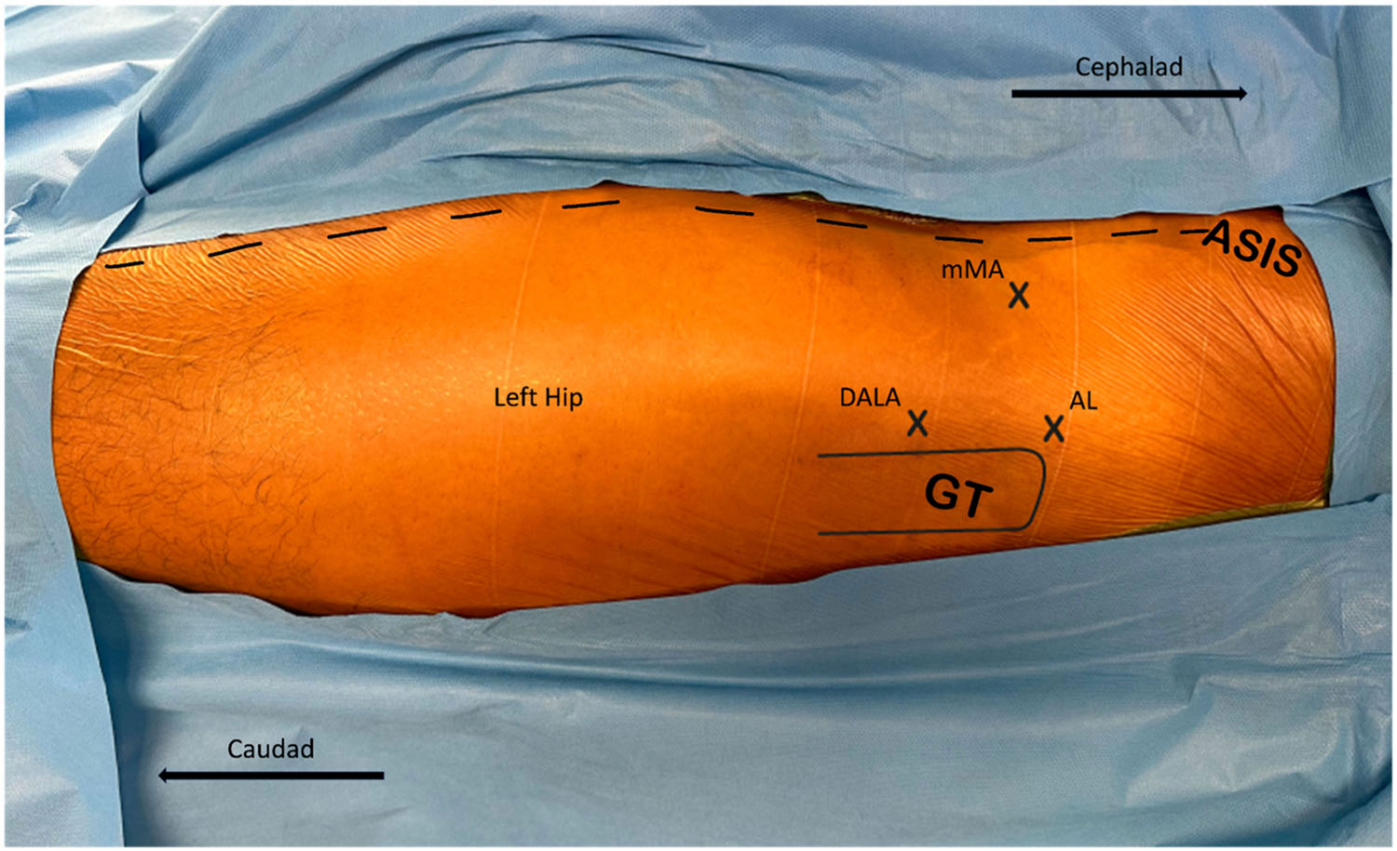
Arthroscopic portal entry points demonstrated on the left hip. Related anatomical landmarks should be marked priorly. AL, antero‐lateral portal; ASIS, anterior superior iliac spine; DALA, distal antero‐lateral accessory portal; GT, greater trochanter; mMA, modified mid‐anterior portal.

The hip joint is located deep in the skin and has a narrower manoeuvre zone. Identification of the anatomic landmarks are crucial because portal accesses will be opened accordingly and used throughout the surgery. First, a line connecting the anterior superior iliac spine (ASIS) and patella was drawn. Crossing this line medially carries a risk for possible vascular damage. Second, a greater trochanter (GT) is palpated and proximal curvature is identified. The anterolateral portal (AL) is marked at the anterosuperior corner of the GT and the modified mid‐anterior portal (mMA) is marked four fingers above and slightly distal to the AL portal. Distal anterolateral accessory portal (DALA) marked distal to the AL portal forms a geometrically bi‐equal triangle connecting the mMA to the AL and DALA portals (Figure 2).

The probability of a surgeon achieving successful outcomes is significantly influenced by the establishment of optimal access points. Improper portal placement led to compromised visibility and hindered the ability to appropriately manipulate the joint throughout the surgical procedure. Proper portal placements are assigned depending on the previous drawings.

**Figure 3:**
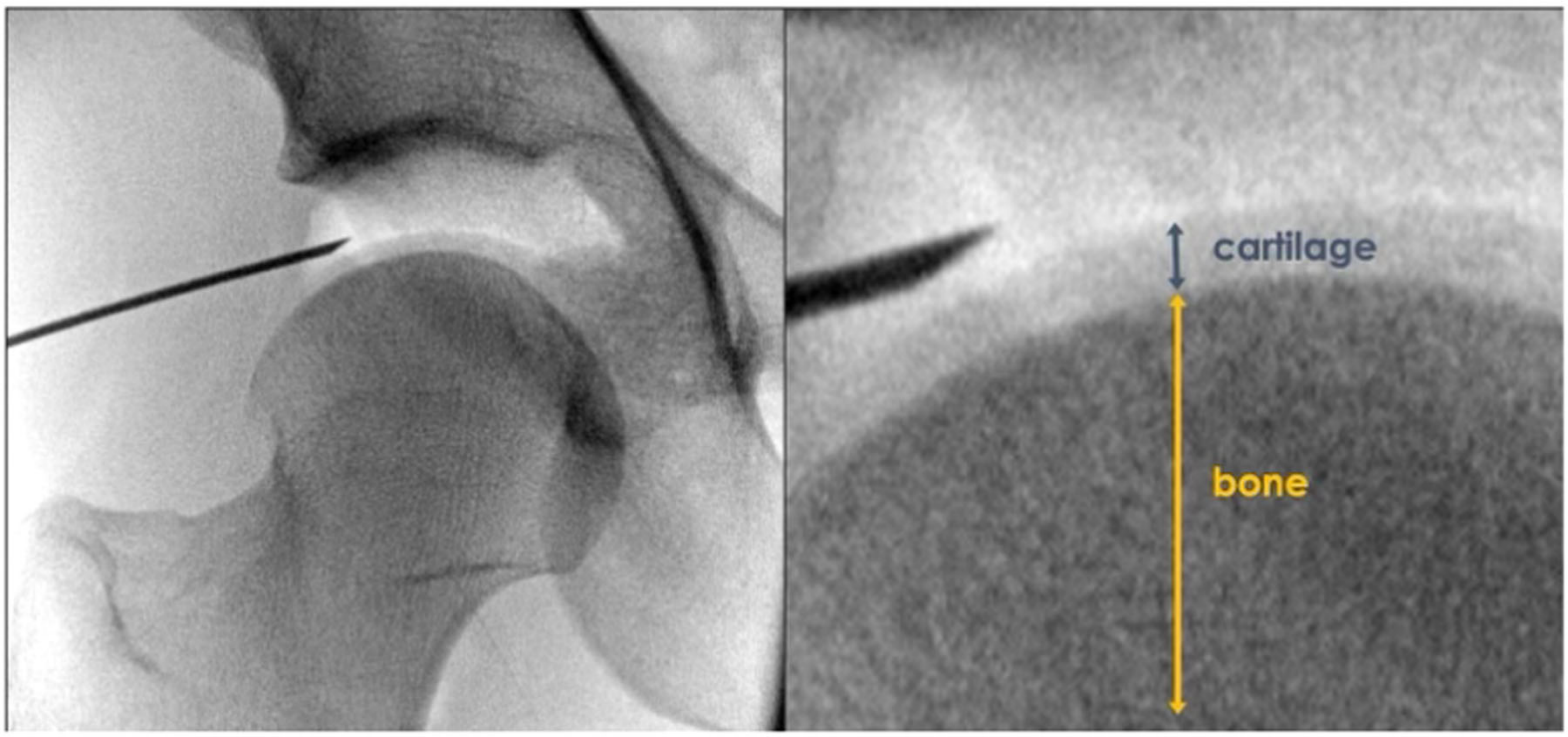
Placement of the needle during first access. Please note that the needle is introduced into the joint slightly further from the bone to avoid iatrogenic chondral and labral damage. Please also note that the optimal trajectory of the needle is parallel to sourcil.

AL portal is located slightly anterior and proximal to the tip of the GT. C‐arm confirmation of the positioning of the guide wire is crucial. If the appropriate entrance point is established, the guide wire lies parallel to the sourcil and the trajectory of the wire points out the deepest point of the acetabulum (12 o'clock) (Figure 3). Then, the scope is introduced to the joint. The mMMA portal opened under direct visualisation of the arthroscopic triangle to display the 2 o'clock position of the acetabulum. It has to be kept in mind that AL and mMA portals will be connected by capsulotomy. Therefore, over‐medialization of the mMA portal may result in an extensively large capsulotomy. DALA portal will open when placing anchor to acetabulum at 1–3 o'clock position in future from interportal capsulotomy. It provides a better drilling angle that prevents penetration to chondral tissue [41].

#### Protips

- AL portal is the initial step and affects the whole flow of the surgery. Do not hesitate to spend some time on the placement and make sure that your guide wire hits the 12 o'clock position.
- Progression of the guidewire to the deepest point of the acetabulum is a minor confirmation that the entrance trajectory is correct and ready for incision.
- Inflation of both the inside and outside of the capsule gives perfect radiographic visualisation and information about its thickness.
- Make sure to make a superficial skin incision for the mMA portal first then make it deep due to its close proximity to the lateral femoral cutaneous nerve.
- For the DALA portal, first place the insertion needle perpendicular to the skin. After that puncture the skin. Then follow the trajectory of the previously placed cannula inside the joint and make it parallel to it. If the trajectories are matched, the insertion needle comes out from the interportal capsulotomy zone and points towards the 1–2 o'clock position, which is a perfect place for anchor placement at the anterior acetabular rim.
- Fluoroscopy or ultrasound may be used to determine the adequacy of traction [44]. We prefer to use fluoroscopy.

### Capsulotomy

**Figure 4:**
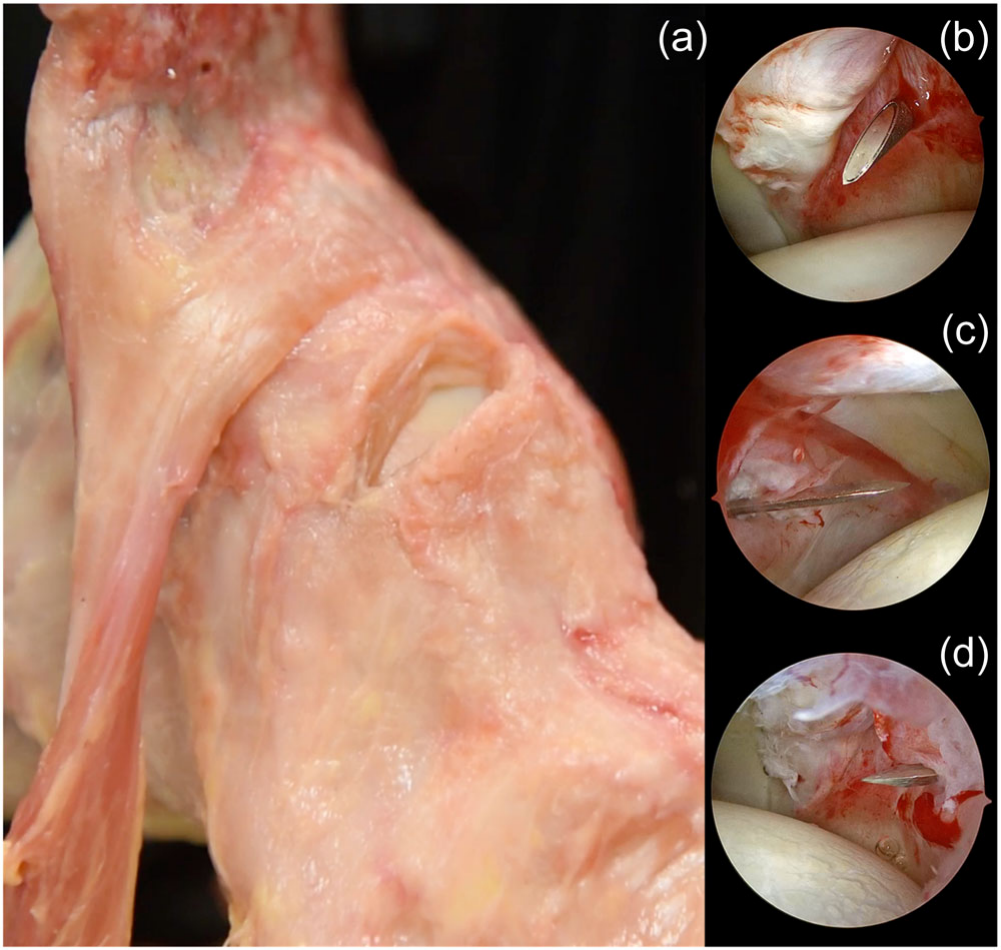
(a) Demonstration of the periportal capsulotomy on a cadaveric specimen. (b) Identification of the modified mid‐anterior (mMA) portal entry point. The guide needle is placed below the labrum without harm to the femoral cartilage. (c) Initial incision is extended towards the scope while performing periportal capsulotomy at the anterolateral (AL) portal while viewing through the mMA portal. (d) Please note that the periportal incision trajectory will act as a precursor for the interportal capsulotomy directed from the mMA to the AL portal while viewing through the AL portal.

**Figure 5:**
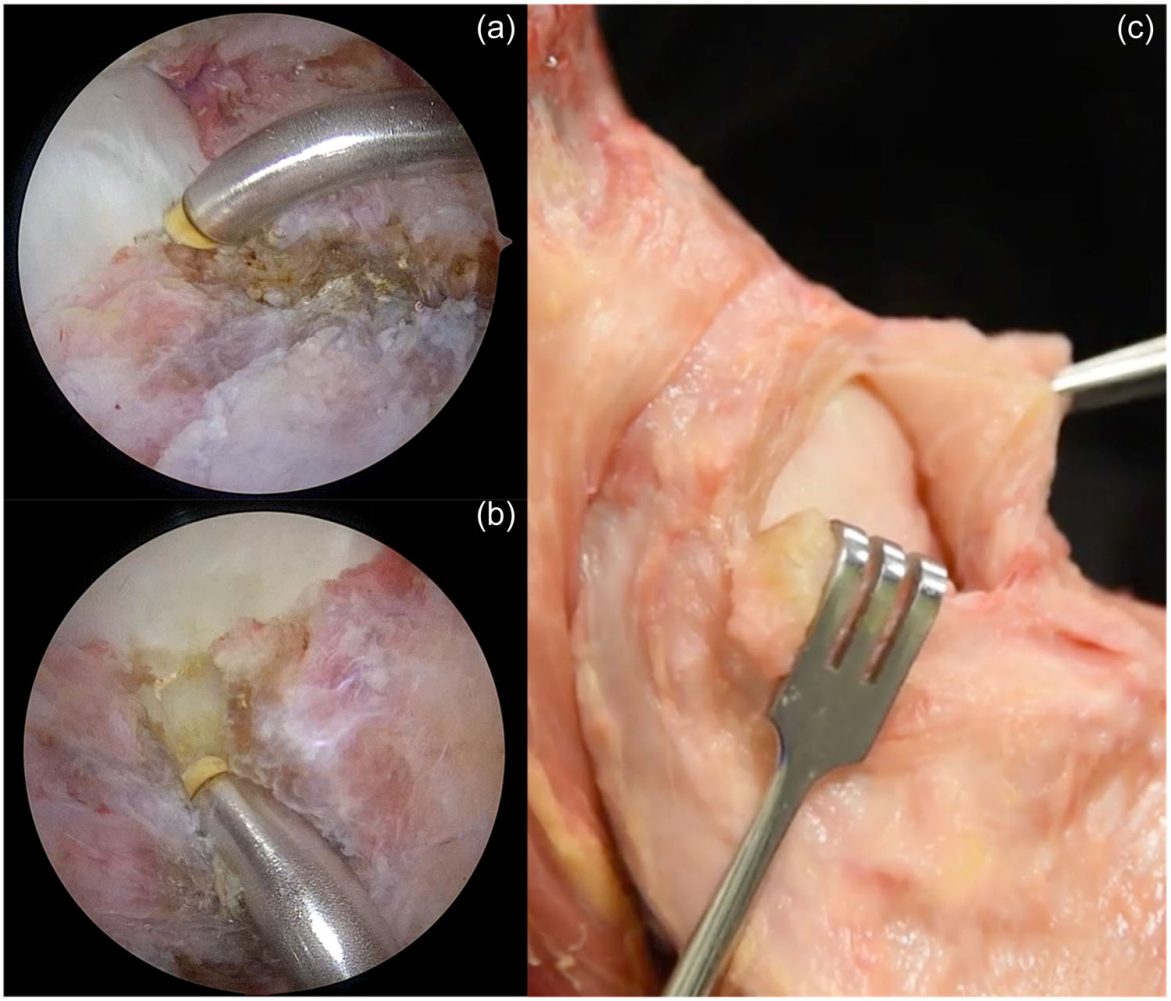
(a) Marking of the direction of the T‐capsulotomy. (b) Completion of the capsulotomy creates two limbs and allows perfect exposure of the CAM lesion. (c) Demonstration of a T‐capsulotomy on a cadaveric specimen.

Capsulotomy is an essential procedure to ensure adequate manoeuvre zone to facilitate both diagnostic and therapeutic interventions. The management of the hip joint capsule is a subject of significant interest, with a considerable amount of debate surrounding the necessary extent of iatrogenic capsular injury for effective access to abnormalities in both the central and peripheral compartments. One of the most common capsulotomy techniques is interportal. One notable benefit associated with this surgical approach is its ability to provide a broader surgical field of view, facilitating the management of deformities in a more convenient manner. However, a major drawback is the substantial damage done to the joint capsule, particularly the iliofemoral ligament. As a consequence, it becomes essential to perform capsule repair. Also, to minimise the risk of possible damage, it is recommended to limit the length of the interportal capsulotomy to 2 cm or less. Once the AL and mMA portals are prepared under fluoroscopy guidance, the scope is switched to the mMA portal to directly visualise the exact location of the AL portal. In periportal capsulotomy, the blade is directed towards the mMA portal. In other words, the direction of the blade should be going towards the camera itself (Figure 4). Even for the competent arthroscopists, orientation inside the joint may be confusing and natural spatial orientation might be misleading. To stay on the safe side do not attempt to perform periportal capsulotomy free hand. After completion of the periportal capsulotomy, the surgeon experiences a loosening sensation and the instruments begin to move freely. The line created with periportal capsulotomy will act as a guide for the interportal capsulotomy. Then, the scope is switched back to the AL portal again and the interportal capsulotomy is completed by following the trajectory of the periportal capsulotomy from mMA to the AL portal direction [15]. Once the interportal capsulotmy is completed the proximal femur and acetabulum are exposed. However, the CAM lesion is still covered with the distal part of the capsule. T‐capsulotomy is performed to expose the head–neck junction of the femur. Interportal capsulotomy acts as the roof of the T‐capsulotomy. Then, a line is marked perpendicular to the roof and cut gently to create two limbs of the capsule. After completion of the T‐capsulotomy clear unobstructed vision is acquired to perform on the CAM lesion (Figure 5).

#### Protips

- As the CAM lesion mostly accumulates at the superolateral border, T‐capsulotomy is a must.
- To prevent microinstability, it is recommended to apply the traction‐assisted interportal technique in patients who fall within the borderline category or have mild CAM deformity mostly at the anterior location.

### Traction stitches

**Figure 6:**
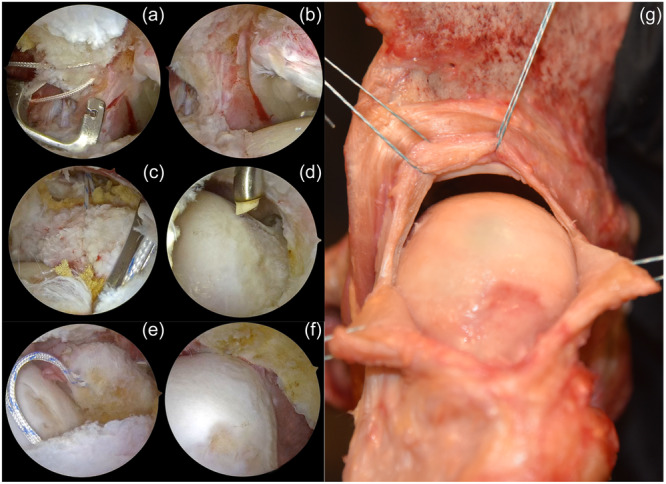
(a) Passing the suture through the capsule to create a working space between the capsule and labrum. (b) Note the extension of the sight after the capsule is elevated. (c) Passing the suture through the distal limb of the interportal capsulotomy to maintain a broader view of the CAM deformity at the femoral head–neck junction without the need for a T‐capsulotomy. (d) Note the extension of the sight after the elevation of the capsule. (e) Passing the suture through the medial and lateral flaps of the T‐capsulotomy to maintain a broader view for CAM resection. (f) Note the extension of the sight after the capsule is elevated. (g) Illustration of the traction stitches applied around the capsule on a cadaveric specimen.

Traction stitches enhance visibility and provide access by widening the operative field within the capsule. Furthermore, it facilitates the establishment of a suitable working area for the labrum and capsule without compromising the thickness of the capsule during acetabuloplasty and advanced pincer resection. Another point to consider is that closure of the capsulotomy after completing all procedures is as important as performing the capsulotomy. To maintain the integrity of the capsular tissue during closure, it is recommended to properly position traction stitches. First, traction stitches are placed in the capsule at the mMA portal and the procedure is repeated after switching the portal. Then traction is applied and the capsule is hung (Figure 6). Limbs of the traction stitches are held in position with a curved hemostat.

#### Protips

- To provide sufficient suspension, traction sutures are applied and subsequently tightened using a hemostat on the skin.
- While performing acetabuloplasty, viewing through the AL portal, a traction stitch is used through the mMA portal while preparing the anterior acetabulum. Likewise, while viewing through the mMA portal and preparing the superolateral part of the acetabulum, traction stitches are used to elevate the capsule.
- Do not hesitate to apply more than one traction stitch to improve visualisation as required.

### Acetabuloplasty

The standard therapy for individuals diagnosed with pincer‐type FAI often involves the surgical procedure of acetabular rim trimming, which aims to remove lesions. During the process of removing excessive bone parts, our aim is to maintain the integrity of the chondrolabral junction (CLJ). This can be achieved by avoiding detachment of the labrum or penetration of the junction. Significant variations in the lateral and anterior centre edge angles, as well as the presence of the crossover sign, are indicative of over‐coverage. These abnormalities suggest the need for arthroscopic resection. The establishment of a preoperative plan is of utmost importance as it enables the correlation of such plans with intraoperative fluoroscopic images and facilitates direct vision during rim excision. One of the critical concerns is to minimise or avoid resection to prevent the risk of under‐coverage in dysplastic hips.

**Figure 7:**
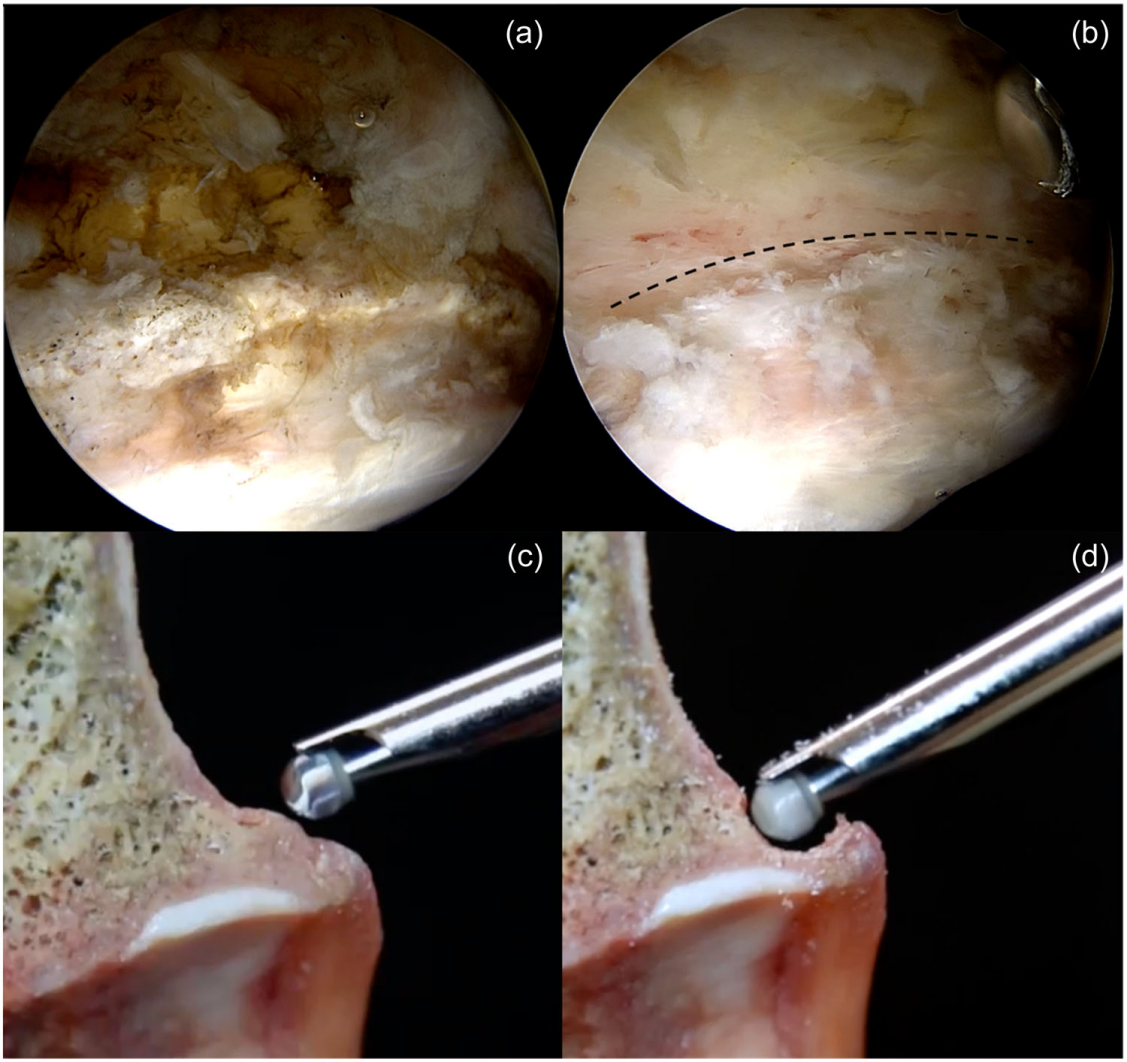
(a) Arthroscopic view of the pincer lesion. (b) Obtained smooth surface after resection. Note the integrity of the chondrolabral junction is maintained. (c–d) Cross‐sectional demonstration of the pincer excision on a cadaveric specimen.

Keeping the CLJ intact during the pincer excision is fundamental. Avoid generating deep resection over the areas and excise the bone layer by layer from 11 o'clock to 3 o'clock preferably. The superolateral part of the acetabulum (around 12 o'clock) is visualised from the AL portal and the burr is introduced from the mMA portal. Excision begins approximately 5 mm superior to CLJ and proceeds towards the labrum. Work meticulously to equalise the natural bony level. Then, the scope is switched to the mMA portal and the same procedure is repeated for 1 o'clock to 3 o'clock zones of the acetabulum. In the end, a smooth and circular surface suitable for anchor placement is obtained (Figure 7).

### Labral repair

**Figure 8:**
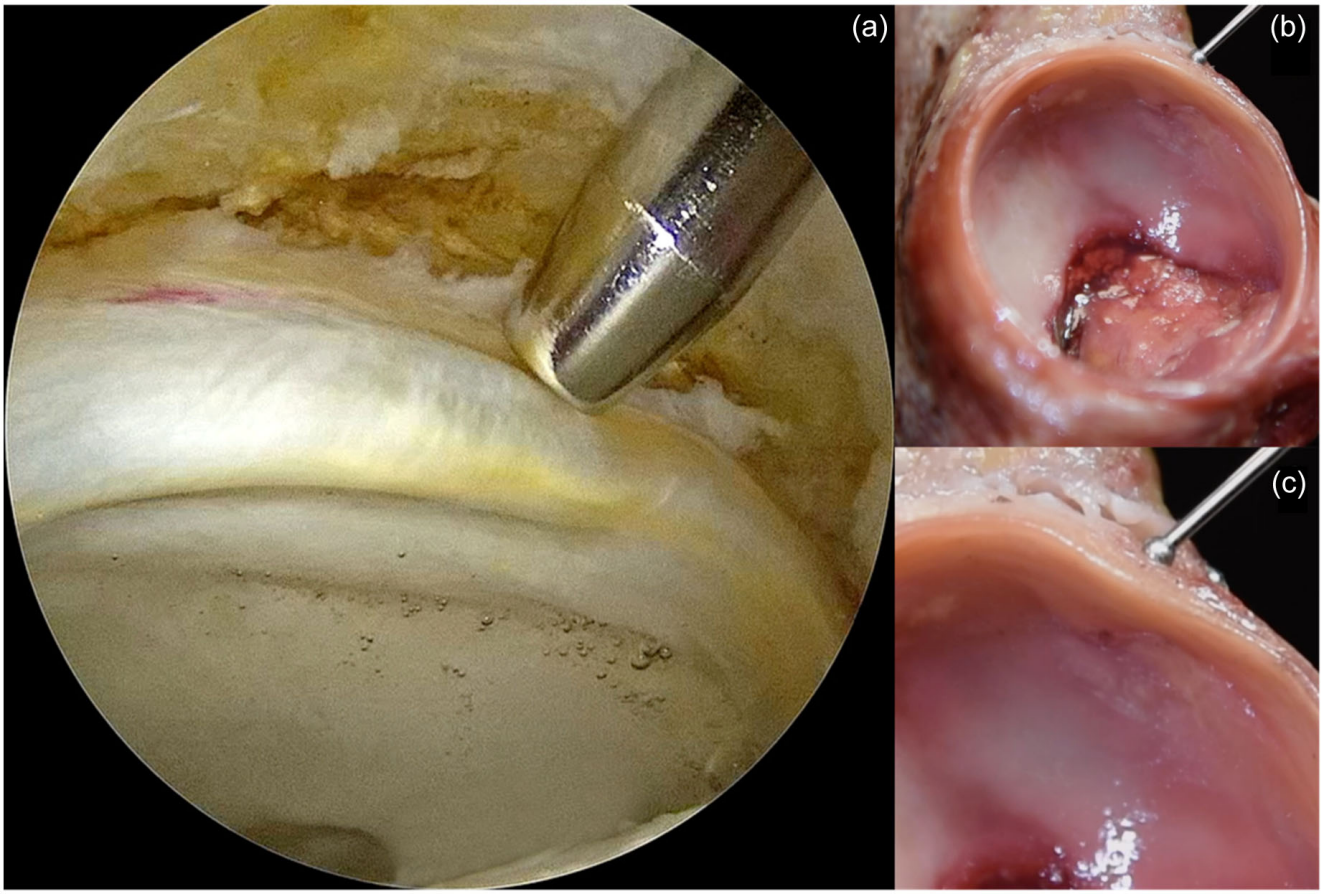
(a) Recognition of the wave sign (Bubble sign). Acetabular cartilage is delaminated due to force transmitted by CAM lesion. (b–c) Demonstration of delamination in a cadaver model.

Congruency of the femoral head and acetabulum is provided by the labrum. The integrity of the hip joint is maintained by the suction force, which is generated and guarded by the labrum as well. In addition, it also increases the contact area and reduces the contact pressure [6]. It has been shown that smaller labrum tissue generates a smaller suction seal force [42]. CLJ is also rich in free nerve endings [1]. In CAM lesions mostly the anterosuperior part of the CLJ contacts with the CAM lesion prematurely causing delamination of the CLJ and provoking pain (Figure 8). Therefore, labral repair alone does not provide adequate protection on chondral tissue [43]. Simultaneous treatment for both pathologies is required to diminish symptoms. Restoration of the CLJ is shown to be associated with superior functional outcomes and restoring the suction seal more successfully than physical therapy, chondral debridement and rim trimming alone [9, 17]. Unique biomechanical properties of the labrum play a major role in the hip joint in terms of stability and endurance. In light of these findings, surgical procedures rapidly evolved to restore the integrity of the acetabular labrum.

FADIR test (flexion, adduction and internal rotation) mimics the premature contact of the CLJ and proximal femur. During the manoeuvre, if the pain is generated, it is indicated as positive and disturbance of CLJ is likely.

There are many different pathologies in the differential diagnosis of groin pain. Due to complex regional anatomy, overlapping of clinical entities frequently occurs. The contribution of FAI to groin pain must be determined before surgery. The intraartricular local anaesthetic administration test is an excellent diagnostic tool to rule out extraarticular pathologies. This diagnostic tool helps identify if the pain is originating from within the joint or from surrounding structures. If the patient experiences significant pain relief after the injection, it suggests that FAI may be the primary cause of their groin pain and surgery may be a suitable treatment option. The patient's benefit from the injection is expected to be almost equivalent to the benefit from surgery.

Labral debridement is unable to fully restore the suction‐seal effect, which results in poor load distribution and increased shear stress [10]. To prevent these potential complications, we prefer to perform labral repair or reconstruction, which is now proven to be superior [17, 25].

**Figure 9:**
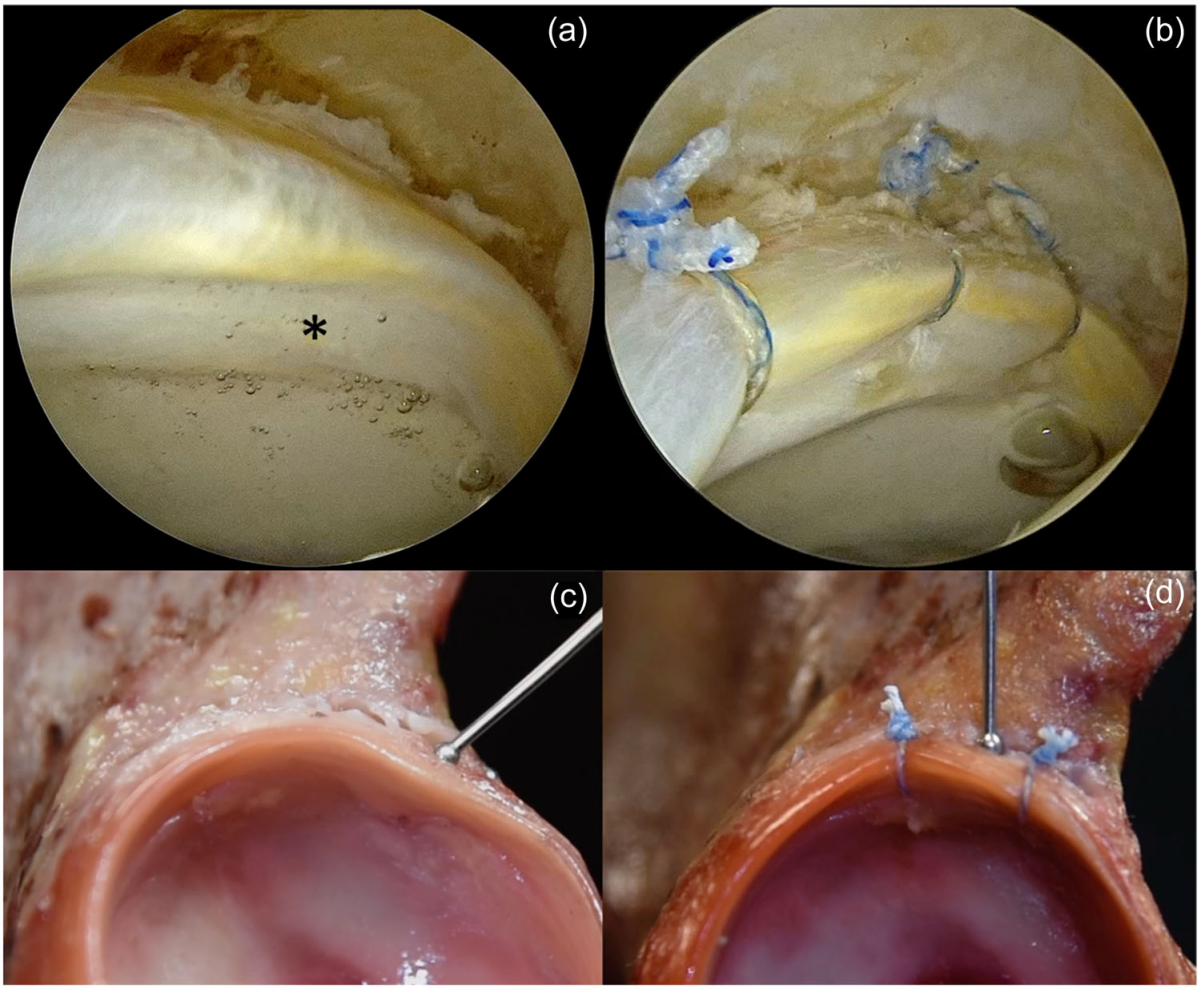
(a) Disturbed chondrolabral junction (CLJ) resulted in an accumulation of fluid behind the cartilage (*: Bubble sign). (b) The lax labrum is addressed with three evenly spaced sutures and repaired. (c) Demonstration of the bubble sign. (d) Please note that the labrum is repaired and the passage is closed.

Once the traction stitches are applied and optimal visualisation is obtained from the AL portal, the drill guide is placed in the 12 o'clock position. The trajectory of the drill guide should be adjusted carefully. Place the scope intraarticularly to show acetabular cartilage and advance the drill gradually. Check the chondral mobility simultaneously to avoid penetration. Then, an anchor is placed and stability is tested with gentle traction. The mobility of the anchor limbs was verified. One limb of the sutures is wrapped around the labrum with the help of a suture passer. Then, two half‐hitches are placed on the post limb (limb at the acetabular side), and a knot is placed at the acetabular rim side of the joint. The second anchor is placed through the DALA portal. It provides a safer drilling angle to place the anchor between 1 and 3 o'clock. A similar procedure is repeated and the labrum is hung to the acetabulum with two to three evenly spaced anchors (Figure 9).

#### Protips

- Anchors should be placed at 12 and 3 o'clock positions and spaced evenly.
- Most posterior and anterior parts of the labrum are relatively thinner and care must be given to avoid rupture during rim trimming and suture passage.
- Curved guide and flexible anchors for 12 o'clock anchor placement to prevent chondral damage.
- Knot should be placed at the acetabular rim side. Preferably at the most acetabular border.

### Osteochondroplasty

Asphericity of the femoral head leads to CAM impingement, which causes a loss of offset at the junction of the femoral head and neck. For patients with hip impingement symptoms, arthroscopic osteochondroplasty may increase the range of motion and reduce discomfort. Osteochondroplasty is a frequently employed therapeutic intervention; yet, attaining optimal osteochondroplasty outcomes might present challenges. The procedure may lead to excessive or insufficient resection. Among the primary causes of failure following FAI correction, residual CAM deformity stands out as the most common [39]. It is important to evaluate the boundaries of the abnormality and this has to be done before the beginning of the initial bone resection [38]. While there is currently no consensus on how to achieve an optimal CAM resection, the senior author of this study expresses a preference for beginning the resection from the distal region and progressing proximally, to fully restore the spherical shape of the femoral head. The resection procedure starts at the anterior region of the femoral head–neck junction, specifically at 30° flexion. Subsequently, the superolateral region is addressed at 0° flexion. Finally, the most anterior region is targeted at greater flexion angles, accompanied by increased external rotation.

The decision of the adequacy of excision by direct arthroscopic view may result in over‐ or under‐excision. To prevent this, the removed part should be confirmed with C‐arm during the resection. Circumferential evaluation of the femoral neck can be achieved via multiple fluoroscopy views. Consecutive intraoperative anteroposterior (AP) views taken when hip in extension and neutral rotation, 30° internal rotation and 30° external rotation; and then when hip in flexion neutral rotation, 40° external rotation and 60° external rotation. These multiple fluoroscopy views allow to evaluate CAM deformity from 11:45 AM to 2:45 PM with computed tomography (CT) equivalent precision [16, 38].

#### Protips

**Figure 10:**
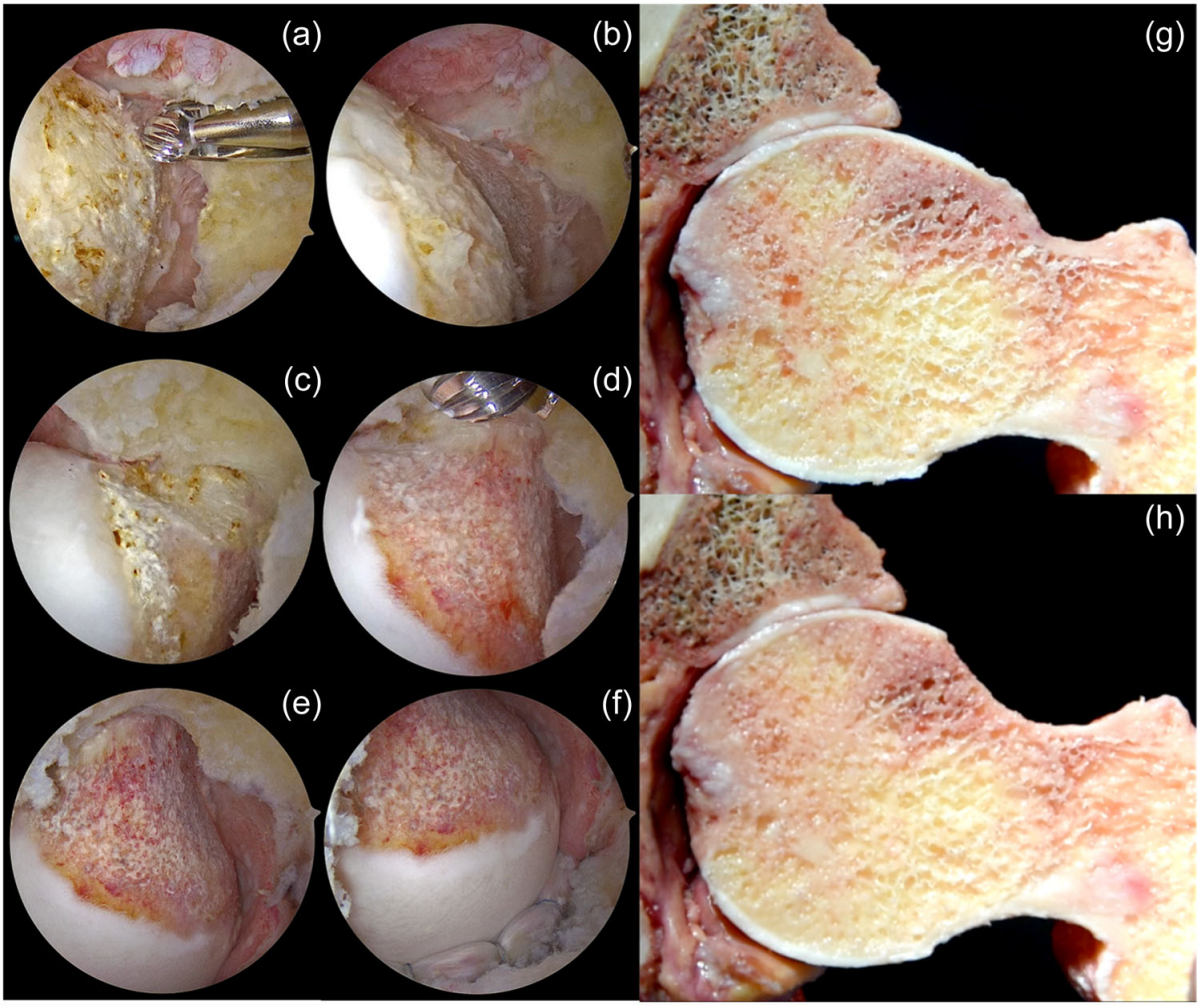
(a) Determination of the CAM lesion in the anterior femoral head–neck junction at 30° flexion, slightly external rotation in a left‐sided patient while viewing through the modified mid‐anterior (mMA) portal. Please note the 5.5 mm burr (Smith & Nephew) pointing to the most distal location of the CAM deformity to determine the starting location of the resection. (b) Beginning the resection from the distal part of the anterior neck region around 1:30 o'clock position. (c) Superolateral part of the CAM lesion in 0° flexion while viewing through the mMA portal. (d) Viewing the superolateral part following the appropriate resection. (e–f) Restoration of the natural curvature of the femoral head–neck junction following the resection. (g) Cross‐sectional demonstration of CAM lesion on a cadaver model. (h) Restoration of the sphericity of the femoral head after CAM resection on a cadaver model.

- To ensure a successful outcome of osteochondroplasty, it is recommended to begin the resection process from the distal region of the CAM lesion. The aim is to restore the full perfect sphericity of the femoral head without attenuation of the head.
- Our preferred approach involves the application of capsular tagging stitches to each limb of the T‐capsulotomy. Subsequently, the traction should be released, followed by the performing of CAM resection with the utilisation of traction stitches. These stitches enhance retraction and visualisation during the process of CAM lesion resection (Figure 10).
- Mark the physical scar with fluoroscopy confirmation. Flex the hip joint 30°. This line usually corresponds 1 cm away from the labrum at 30° flexion and indicates the terminal point for the resection.

### Capsule closure

The hip joint's stability and mobility are influenced by the capsular ligaments, including the pubofemoral, ischiofemoral, and iliofemoral [45]. Among the ligaments examined, the iliofemoral ligament emerges as the primary contributor to external rotation [28] and demonstrates its efficacy in reducing anterior instability of the hip [20, 21]. Therefore, it is crucial to retain the integrity of these structures and execute the capsulotomy with caution, since the scarifying of these ligaments might result in significant anterior displacement of the hip [4].

On the other hand, the term hip instability, even frequently announced; lacks objective critera to define precisely. In addition, it is an ill‐defined term and demonstrates poor clinical relevance. In one way or another, it is established that capsular laxity is linked to instability, with particular emphasis on the anterior capsule acting as a restraint against excessive motion [19, 24]. Consistent with biomechanical studies, recent literature demonstrated superior patient‐related outcome scores with capsule closure [33, 37]. In addition, prior studies have also demonstrated that the closure of the joint capsule during arthroscopy has the potential to protect against instability, reduce the need for subsequent surgical revisions and enhance postoperative outcomes [4, 12, 23], and a lesser arthroplasty conversion rate was also observed in long term [37]. Consequently, with a quicker return to athletic activities, and a diminished degree of anxiety over hip subluxation, dislocation or other less obvious instabilities, the closure of the hip capsule becomes an even more significant issue to be taken into account.

Especially in the case of individuals who were diagnosed with borderline hip dysplasia, the importance of the hip capsule becomes more prominent for the maintenance of hip stability. When the lateral centre edge angle (LCEA) is between 20–25°, the success of the capsular plication becomes the best predictor for better functional outcomes [2]. In line with the literature, the closure of the capsule more effectively restores native hip biomechanics. Therefore, we prefer complete closure of the capsule in all cases.

#### Protips

**Figure 11:**
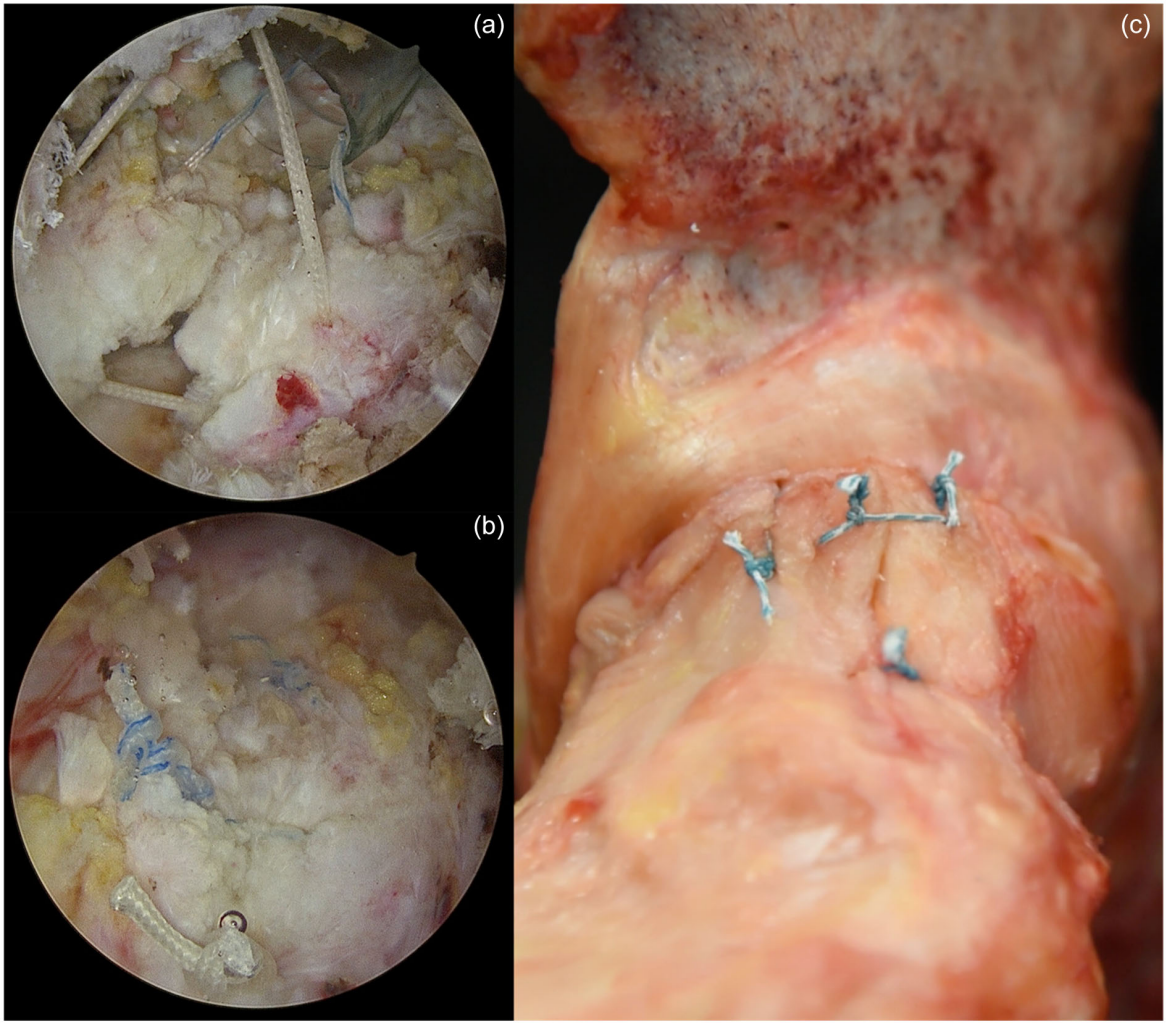
(a) Arthroscopic view of the interportal capsulotomy. Sutures are passed to the corresponding parts of the capsule flaps. (b) Watertight closure of the capsule after the knots are tied. (c) Demonstration of the capsule closure in a cadaver model.

- Once the CAM resection is terminated, use a suture passer to carry one limb of the previously placed traction sutures to the reciprocal flap of the capsule. Hence you can complete capsule closure with minimal effort (Figure 11).
- We highly recommend considering capsule repair or even plication following hip arthroscopy, particularly in patients with lower acetabular coverage and those exhibiting additional instability criteria.
- Following capsule closure, insert a spinal needle into the joint to administer a local anaesthetic, ensuring improved pain control immediately after surgery.

### Expert opinion

In recent times, hip arthroscopy has gained in popularity. It has a significant learning curve and demands considerable dedication. Surgical apprenticeship contributes markedly to the educational experience. Every component of the operation, from setting up the traction table, securing the feet and positioning the patient during the procedure, to closing the capsule, requires distinct protocols that should be standardised. Particular instruments designed for hip arthroscopy eliminate repetitive motions, thereby preventing iatrogenic damage and complications while decreasing the overall duration of the operation. Contemporarily, capsule management, capsule closure and traction stitches are the keystones of successful hip arthroscopy. In the near future, hip arthroscopy will be gaining an exponential amount of attention.

## AUTHOR CONTRIBUTIONS

Safa Gursoy, Yigit Umur Cirdi and Muge Kirac prepared the manuscript and collected intraoperative pictures.  Safa Gursoy and Yigit Umur Cirdi made the critical revision and searched related literature. Safa Gursoy and Jorge Chahla shaped the final structure of the manuscript. All authors in this study were fully involved in the study and preparation of the manuscript and the material within has not been and will not be submitted for publication elsewhere.

## CONFLICT OF INTEREST STATEMENT

Jorge Chahla is a board or committee member in American Orthopaedic Society for Sports Medicine and a paid consultant in Arthrex, Inc., he is a board or committee member in Arthroscopy Association of North America and a paid consultant in CONMED Linvatec, he is a board or committee member in International Society of Arthroscopy, Knee Surgery, and Orthopaedic Sports Medicine. He is a paid consultant in Ossur, he is a paid consultant and paid presenter or speaker in Smith&Nephew. The remaining authors declare no conflict of interest. Authors are permitted to reproduce material from other sources. No clinical trial registration present for expert opinions.

## ETHICS STATEMENT

In accordance with federal regulations, this research study has been determined to be exempt from Institutional Review Board (IRB) oversight. The exemption status is based on the nature of the research, which involves no risk to participants and does not involve sensitive or personally identifiable information. Images such as X‐rays, laparoscopic images, ultrasound images, brain scans and pathology slides, unless there is a concern about identifying information, do not require informed consent. All pictures in the manuscript were taken from the personal archive of the Safa Gursoy M.D and do not involve any sort of identifying personal information.

## Data Availability

The authors confirm that the data supporting the findings of this study are available within the article.
